# BRCA2 affects the efficiency of DNA double-strand break repair in response to N-nitroso compounds with differing carcinogenic potentials

**DOI:** 10.3892/ol.2013.1269

**Published:** 2013-03-22

**Authors:** WEN-TING ZHAO, YU-TIAN WANG, ZHAO-WEI HUANG, JING FANG

**Affiliations:** 1 Key Laboratory of Nutrition and Metabolism, Institute for Nutritional Sciences, SIBS, Chinese Academy of Sciences, Shanghai 200031;; 2 Department of Gastroenterology, Changzheng Hospital, The Second Military Medical University, Shanghai 200003, P.R. China

**Keywords:** DNA double strand breaks, BRCA2, N-nitroso compounds, DNA damage repair

## Abstract

The tumor suppressor gene breast cancer susceptibility gene 2 (BRCA2) is frequently mutated or epigenetically repressed in human cancer and has a significant role in the homologous recombination (HR) of DNA double-strand breaks (DSBs). Although N-nitrosodiethylamine (NDEA), N-nitrosodiethanolamine (NDELA) and N-nitrosodipropylamine (NDPA) have similar chemical structures and are able to induce DNA damage, they have varying carcinogenic risks. We hypothesized that the DNA damage repair pathways that are induced by these N-nitroso compounds (NOCs) may differ and that this may contribute to the genotoxic-carcinogenic effect of the NOCs. The present study aimed to characterize the formation of DSBs by NDEA, NDELA and NDPA and also to investigate whether BRCA2 is involved in the DNA damage response. The NOCs were observed to time-dependently induce DSBs and the expression of γ-H2AX in gastric cancer SGC7901 cells. It was observed that the DNA damage induced by NDEA, the most potent carcinogen, was not repaired as efficiently as that caused by NDELA or NDPA. The expression of BRCA2 and RAD51 was demonstrated to be inhibited by NDEA treatment but upregulated by NDELA or NDPA treatment. Furthermore, the knock down of BRCA2 expression impaired the DNA damage repair induced by NDELA or NDPA. The cells with this knock down exhibited an increased sensitivity to NDELA or NDPA treatment, but not to NDEA. These findings suggest that a BRCA2-mediated pathway contributes to differential DSB repair and sensitivity in response to NOC exposure and that it may be associated with the genotoxic-carcinogenic potential of NOCs.

## Introduction

Tumor suppressor breast cancer susceptibility gene 2 (BRCA2) is responsible for a large percentage of familial breast cancer cases (1,2). In addition to breast cancer, BRCA2 mutations are also linked to other types of cancer, including ovarian, hepatocellular, pancreatic, prostate and gastric tumors (3–5). The protein encoded by this gene is involved in the repair of chromosomal damage and has an essential role in the repair of DNA double-strand breaks (DSBs) through homologous recombination (HR) (6–8). In support of this theory, mammalian cells lacking functional BRCA2 have been shown to be sensitive to DNA damaging agents (9,10), exhibit genomic instability (11–13) and are deficient in homology-directed DNA repair (8,14).

BRCA2 interacts with a number of DNA repair proteins, including γ-H2AX and RAD51 (15–17). γ-H2AX foci formation functions to recruit DNA repair factors to the damaged sites, enforcing the HR of DNA DSBs and linking the process of chromatin remodeling to DNA repair (16,18,19). RAD51 is a DNA recombinase that is essential in initiating the HR process by mediating DNA strand exchange during recombination. BRCA2 is required for RAD51 foci assembly in response to ionizing radiation (IR)-induced DNA DSBs (15,17,20,21).

N-nitroso compounds (NOCs) and their precursors exist extensively in the environment, certain occupational settings, diets, tobacco products, cosmetics and pharmaceutical products, and are endogenously formed in the human body from dietary components (22,23). Many NOCs have been identified as carcinogenic (23–27) and the International Agency for Research on Cancer (IARC) has classified four NOCs as probably carcinogenic to humans and another 15 as possibly carcinogenic (23,28–30). The carcinogenic effect of NOCs is usually attributed to their DNA damaging and genotoxic properties (23,31,32).

Certain studies have shown that NOCs are able to induce DNA single-strand breaks and DSBs (31,32), suggesting that DNA repair by the HR pathway may function in repairing the DNA damage induced by NOCs. However, there have been no studies on the role of HR in the repair of DNA damage induced by NOCs. We hypothesized that, as a DNA damage response, the BRCA2-mediated HR pathway may be involved in DNA damage repair induced by the NOCs and that this may contribute to their carcinogenic effect. Three NOCs, N-nitrosodiethylamine (NDEA), N-nitrosodiethanolamine (NDELA) and N-nitrosodipropylamine (NDPA), with similar chemical structures and varying carcinogenic risks, which were classified into differing carcinogenic classes in humans according to the IARC, were investigated in the present study (23,24,28–30). The aim of the present study was to characterize the formation and repair of the DNA damage caused by NDEA, NDELA and NDPA in gastric cancer SGC7901 cells and investigate whether BRCA2 was involved in the DNA damage response to these NOCs.

## Materials and methods

### Cells and reagents

Epidemiological studies indicate that NOCs are positively associated with stomach cancer, therefore, the human gastric cancer SGC7901 cell line was used in the present study. The SGC7901 cell line was established from an untreated patient with progressive adenocarcinoma of the stomach. The cells were cultured in Dulbecco’s modified Eagle’s medium (DMEM) with 10% fetal bovine serum. The SGC7901 cells stably transfected with the vector and BRCA2 siRNA (siBRCA2) were cultured in DMEM containing 200 *μ*g/ml of G418 (Invitrogen, Carlsbad, CA, USA). The NDEA, NDELA, NDPA and dimethyl sulfoxide (DMSO) were purchased from Sigma (St. Louis, MO, USA). All chemicals and solvents were of the highest grade commercially available. The NOCs were dissolved in sterile DMSO (0.1%) and freshly prepared each time prior to use. Logarithmically growing SGC7901 cells were treated with NOCs at appropriate concentrations where indicated.

### Comet assay

The comet assay (Trevigen, Inc., Gaithersburg, MD, USA) was performed as described previously, using neutral conditions to detect the DSBs (33). In brief, the cells were harvested, washed with ice-cold PBS and combined with molten LMP agarose, then 75 *μ*l (500–1,000 cells) was immediately added to the comet slide. Subsequent to being hardened, the slides were incubated for 30 min in lysis solution at 4°C, then rinsed with 1X Tris/borate/EDTA prior to electrophoresis for 60 min at 30 V. The slides were rinsed with distilled H_2_O, placed in 70% ethanol for 10 min and then air-dried. To visualize the DNA, 50 *μ*l of a 1:1,000 dilution of SYBR Green (Molecular Probes, Invitrogen) in PBS was added to each slide. The slides were visually scored using fluorescence microscopy (Leica DMS 4000B; Leica, Mannheim, Germany). The comet tail to head ratios or tail lengths were determined using the software package ‘Comet Assay II’ (Perceptive Instruments, Haverhill, Suffolk, UK). A minimum of 50 cells per experiment were analyzed. All the experiments were performed at least three times independently and in triplicate.

### Western blotting

The cells were harvested and lysed with a lysis buffer containing 50 mM Tris-HCl (pH 7.4), 150 mM NaCl, 1 mM MgCl_2_, 100 *μ*g/ml phenylmethylsulfonyl fluoride and 1% Triton X-100 for 30 min on ice. Total cellular extracts (50 *μ*g) were separated by SDS-PAGE and transferred onto nitrocellulose membranes. The membranes were probed with specific primary antibodies, followed by incubation with IRDye680-conjugated secondary antibodies (Rockland, Inc., Gilbertsville, PA, USA). Detection was performed using an Odyssey IR imaging system (LI-COR Biotechnology, Lincoln, NE, USA). The following antibodies were used for the immunoblotting studies: mouse anti-γ-H2AX (serine 139; Upstate, Charlottesville, VA, USA), mouse anti-BRCA2 (Cell Signaling Technology, Beverly, MA, USA), rabbit polyclonal anti-RAD51 (Santa Cruz Biotechnology, Inc., Santa Cruz, CA, USA) and anti-β-actin (Sigma).

### Immunofluorescence studies

The cells were plated onto coverslips and treated with 1X IC_50_ concentrations of NDEA, NDELA and NDPA (0.76, 1.09 and 0.39 mM, respectively) for 1 h. The cells were then fixed and stained with monoclonal anti-γ-H2AX (Upstate). Subsequent to being stained with Alexa Fluor 488-conjugated goat anti-mouse secondary antibodies (Invitrogen), the slides were mounted with Vectashield mounting medium (Vector Laboratories, Burlingame, CA, USA) containing 5 ng/ml 4′,6-diamidino-2-phenylindole (DAPI; Vector Laboratories). The staining images were captured using fluorescence microscopy (Leica DMS 4000B) and a Spot digital camera (Spot Imaging Solutions, Sterling Heights, MI, USA).

### Clonogenic survival assay

To determine the cytotoxicity and IC_50_ concentrations of NDEA, NDELA and NDPA, a clonogenic survival assay was performed on 60-mm cell culture dishes as described previously (34). The SGC-7901 cells were treated with various concentrations of NDEA, NDELA or NDPA for 1 h, followed by drug-free incubation for 10 days. The colonies were stained with crystal violet and counted if ≥50 cells were present. The IC_50_ concentration was calculated as the concentration of NDEA, NDELA or NDPA that killed 50% of the untreated control colonies.

### Small interfering RNA (siRNA) transfection assays

BRCA2 siRNA (SiBRCA2) oligos containing the target sequences of 5′-AAGACACGCTGCAACAAAGCA-3′ were designed and synthesized. Following annealing, the double-stranded siBRCA2 fragment was inserted into a pSilencer 2.1-U6-neo vector (Ambion, Austin, TX, USA) and transfected into the SGC7901 cells with Lipofectamine-2000 (Invitrogen). The pSilencer 2.1-U6-neo vector containing a scrambled sequence (Ambion) was transfected as a nonspecific control. Stable cell lines were established by performing selection in a medium containing G418.

### Statistical analyses

Images of 50 randomly selected cells were evaluated per treatment and the test was performed three times. The Student’s t-test was used to provide the statistical comparisons and P<0.05 was considered to indicate a statistically significant difference.

## Results

### NDEA, NDELA and NDPA-induced DNA DSBs

**Figure 1:**
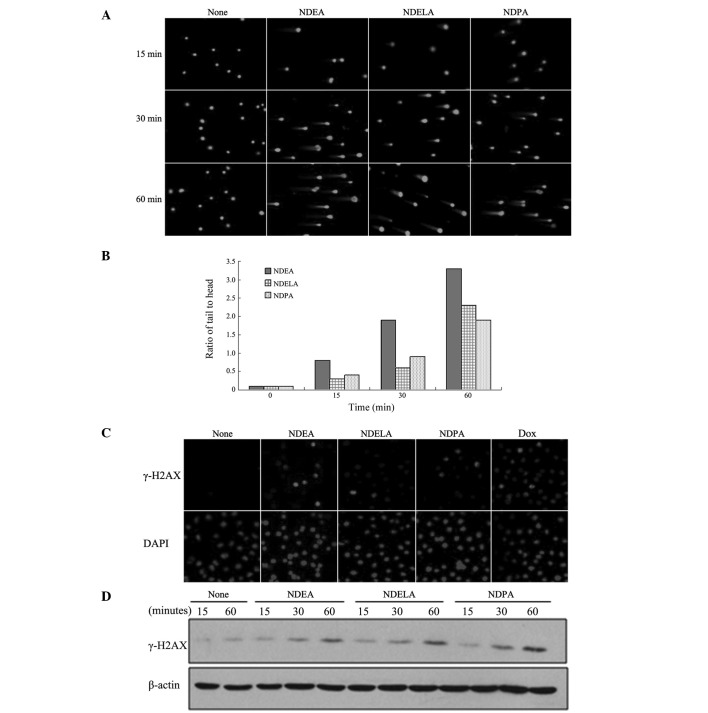
NDEA, NDELA and NDPA-induced DNA double-strand breaks (DSBs). SGC-7901 cells were treated with 1X IC_50_ concentrations of NDEA (0.76 mM), NDELA (1.09 mM) or NDPA (0.39 mM) for 15, 30 and 60 min. (A) DNA damage was determined using a neutral comet assay. Three compounds induced apparent DNA damage in SGC7901 cells. (B) The 50 cells per slide from (A) were analyzed to calculate the tail to head ratio of the SGC-7901 cells. Three compounds induced a time-dependent increase in extent of DNA damage. (C) The SGC-7901 cells were treated with 1X IC_50_ concentrations of NDEA (0.76 mM), NDELA (1.09 mM) or NDPA (0.39 mM) for 1 h. Following treatment, the cells were stained with antibodies against γ-H2AX and counter-stained with DAPI. The cells treated with Dox (1 mM) for 1 h were used as positive controls. NOC treatment lead to formation of γ-H2AX. (D) The SGC-7901 cells were treated with 1X IC_50_ concentrations of NDEA (0.76 mM), NDELA (1.09 mM) or NDPA (0.39 mM) for 15, 30 and 60 min. Following the treatment, the cells were collected and the cellular protein was prepared for western blotting using antibodies against γ-H2AX. Three compounds induced expression of γ-H2AX. NDEA, N-nitrosodiethylamine; NDELA, N-nitrosodiethanolamine; NDPA, N-nitrosodipropylamine; DAPI, 4′,6-diamidino-2-phenylindole; Dox, Doxorubicin.

First, the effects of NDEA, NDELA and NDPA on the DNA damage in the SGC7901 cells were examined. DNA damage was evaluated using the comet assay under neutral electrophoresis conditions to predominantly detect the DSBs. Logarithmically growing SGC-7901 cells were treated with 1X IC_50_ concentrations of NDEA (0.76 mM), NDELA (1.09 mM) or NDPA (0.39 mM) for 15, 30 and 60 min. The comet assays revealed that NDEA, NDELA and NDPA induced apparent DNA damage in the SGC7901 cells as evidenced by the presence of DNA comet tails (Fig. 1A). Analysis of the tail to head ratio, which reflected the degree of DNA damage in the cells, showed that each of the three compounds induced a time-dependent increase in the extent of the DNA damage (Fig. 1B).

Upon the induction of a DSB, the histone variant, H2AX, is rapidly phosphorylated (γ-H2AX) and forms discrete nuclear foci. γ-H2AX foci formation also allows the sensitive detection of DSBs (16,18,35,36). To further confirm that NOCs induce the generation of DSBs, the SGC-7901 cells were treated with 1X IC_50_ concentrations of NDEA (0.76 mM), NDELA (1.09 mM) or NDPA (0.39 mM) for 1 h and then immunofluorescently stained using γ-H2AX antibodies. An examination of the results showed that the NOC treatment led to the formation of γ-H2AX (Fig. 1C), thus indicating the presence of DSBs. Immunoblotting analysis further demonstrated that NDEA, NDELA and NDPA induced the expression of γ-H2AX in a time-dependent manner (Fig. 1D).

### Differential efficiency of DNA damage repair in response to NDEA, NDELA and NDPA treatment

**Figure 2:**
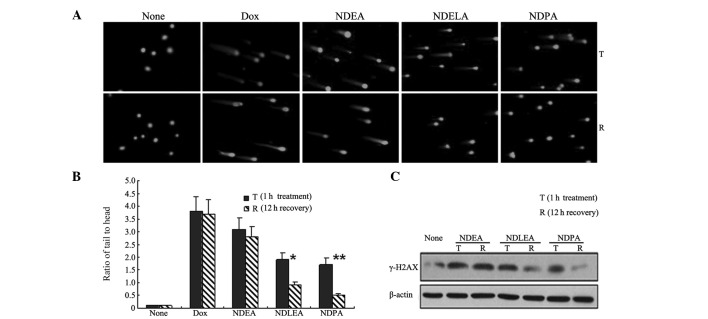
Differential efficiency of DNA repair in response to NDEA, NDELA and NDPA treatment. The SGC7901 cells were treated with 1X IC_50_ concentrations of NDEA (0.76 mM), NDELA (1.09 mM) or NDPA (0.39 mM) for 1 h. The cells were then incubated in a drug-free medium for 12 h to allow DNA repair. (A) DNA damage was detected by neutral comet assays. Dox (1 mM) treatment was used as a positive control. The tail to head ratio of comet DNA in cells treated with NDELA or NDPA was reduced. (B) The 50 cells per slide from (A) were analyzed by measuring the tail to head ratio. ^*^P<0.05 and ^**^P<0.01, T group vs. R group. The tail to head ratio of comet DNA in cells treated with NDELA or NDPA was significantly reduced. (C) The SGC7901 cells were treated with 1X IC_50_ concentrations of NDEA (0.76 mM), NDELA (1.09 mM) or NDPA (0.39 mM) for 1 h. The cells were then incubated in drug-free medium for 12 h. The level of γ-H2AX was determined by western blotting. The expression of γ-H2AX in SGC7901 cells treated with NDELA or NDPA was significantly reduced. NDEA, N-nitrosodiethylamine; NDELA, N-nitrosodiethanolamine; NDPA, N-nitrosodipropylamine; Dox, Doxorubicin.

The comet assay and the data on the expression of γ-H2AX demonstrated that the three compounds, NDEA, NDELA and NDPA, were able to induce a time-dependent increase in the extent of DNA damage. These observations indicated that these three NOCs similarly induced the generation of DSBs. As with the varying carcinogenic potentials, it was unclear whether these NOCs induced similar DNA repair. We hypothesized that the repair mechanism of these NOC-induced DSBs may be different. To examine this hypothesis, the SGC7901 cells were treated with 1X IC_50_ concentrations of NDEA (0.76 mM), NDELA (1.09 mM) or NDPA (0.39 mM) for 1 h, followed by a 12-h drug-free incubation to allow DNA repair. The formation and resolution of the DNA damage were then analyzed using the comet assay. Following a drug-free incubation of 12 h, the tail to head ratio of the comet DNA in the cells treated with NDELA (P<0.05) or NDPA (P<0.01) was observed to be significantly reduced (Fig. 2A and B). However, there was no apparent reduction in the tail to head ratio of the comet DNA in the cells treated with NDEA (P>0.05). The levels of γ-H2AX expression were also studied and it was observed that following a drug-free incubation of 12 h, there was a significant reduction in the expression of γ-H2AX in the SGC7901 cells treated with NDELA or NDPA (Fig. 2C). However, there was no clear change in the γ-H2AX levels in the cells treated with NDEA. These observations suggested that the DSBs induced by NDELA or NDPA were more effectively repaired than those induced by NDEA.

### BRCA2-mediated HR contributes to the differential repair of NOC-induced DSBs in SGC7901 cells

**Figure 3:**
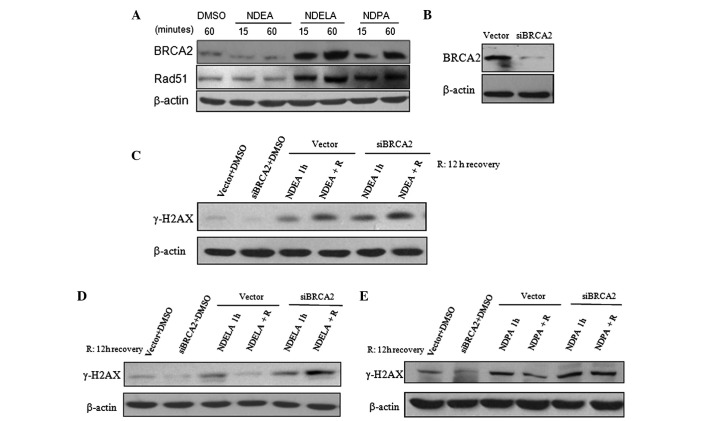
Involvement of the BRCA2-mediated pathway in the DNA repair of NOC-induced damage. (A) The SGC7901 cells were treated with 1X IC_50_ concentrations of NDEA (0.76 mM), NDELA (1.09 mM) or NDPA (0.39 mM) for 15, and 60 min. Following an incubation in drug-free medium for 12 h, the cells were harvested and the expression of BRCA2 and RAD51 was determined by western blotting. The expression of BRCA2 and RAD51 was reduced in SGC7901 cells treated with NDEA, but were notably upregulated in cells treated with NDELA or NDPA. (B) Expression of BRCA2 was knocked down in siBRCA2-transfected SGC7901 cells. BRCA2-targeted siRNA and a control vector plasmid were stably transfected into the SGC7901 cells. (C–E) Differential expression levels of γ-H2AX induced by NOCs in vector- and siBRCA2-transfected cells following a 12-h drug-free incubation. The vector- and siBRCA2-transfected SGC7901 cells were treated with 1X IC_50_ concentrations of (C) NDEA, (D) NDELA and (E) NDPA for 1 h, followed by a 12-h drug-free incubation. The expression levels of of γ-H2AX were determined. The DNA damage induced by NDELA or NDPA was not repaired as effectively as in the vector cells. DMSO (1 mM) treatment was used as a negative control. BRCA2, breast cancer susceptibity gene 2; NOC, N-nitroso compound; NDEA, N-nitrosodiethylamine; NDELA, N-nitrosodiethanolamine; NDPA, N-nitrosodipropylamine; siRNA, small interfering RNA.

In order to determine whether HR was involved in the repair of NOC-induced DSBs, the expression levels of BRCA2 and RAD51, two key proteins in HR (4,8,20), were observed in response to the NOC treatment. The SGC7901 cells were treated with 1X IC_50_ concentrations of NDEA, NDELA and NDPA for 15 and 60 min. The expression of BRCA2 and RAD51 was reduced in the SGC7901 cells treated with NDEA (Fig. 3A). However, the expression levels of BRCA2 and RAD51 were notably upregulated in a time-dependent manner in the cells treated with NDELA or NDPA (Fig. 3A). The results suggest that the BRCA2-RAD51-mediated HR pathway may be significant in the DNA damage repair induced by NDELA and NDPA.

The role of BRCA2 in the repair of NOC-induced DNA damage was further investigated. A BRCA2-targeted siRNA was designed (as described in the Materials and methods) and stably transfected into the SGC7901 cells. The effectiveness of the siBRCA2 construct in knocking down the endogenous BRCA2 level was demonstrated by western blotting. As shown in Fig. 3B, the expression of BRCA2 was effectively knocked down using BRCA2 siRNA transfected into the SGC7901 cells. The control group consisted of SGC7901 cells stably transfected with the control vector plasmid. The vector- and siBRCA2-transfected SGC7901 cells were treated with 1X IC_50_ concentrations of NDEA, NDELA or NDPA for 1 h, followed by a 12-h drug-free incubation. Similar to the cells treated with NDEA (Fig. 3C), the level of γ-H2AX expression in the BRCA2-knockdown cells treated with NDELA (Fig. 3D) or NDPA (Fig. 3E) was not reduced to the level of the vector cells following the drug-free incubation. The results showed that in the BRCA2-knockdown cells, the DNA damage induced by NDELA or NDPA was not repaired as effectively as in the vector cells. This also suggested that BRCA2 contributes to the variations in DNA repair in response to these NOC-induced DSBs.

### BRCA2 confers sensitivity to NOC treatment

**Figure 4:**
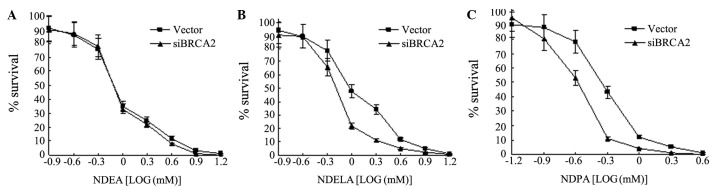
BRCA2 confers sensitivity to NOC treatment. The sensitivity to (A) NDEA, (B) NDELA and (C) NDPA was determined using a clonogenic survival assay in vector- and siBRCA2-transfected SGC7901 cells. BRCA2, breast cancer susceptibity gene 2; NOC, N-nitroso compound; NDEA, N-nitrosodiethylamine; NDELA, N-nitrosodiethanolamine; NDPA, N-nitrosodipropylamine; siBRCA2, small interfering BRCA2 RNA.

Based on the previous observations that BRCA2 is significant for the repair of DNA damage induced by NOCs, we hypothesized that BRCA2 may affect the sensitivity to NOCs. To determine whether BRCA2 affects the sensitivity to NDEA, NDELA and NDPA, a clonogenic survival assay was performed. The vector- and siBRCA2-transfected SGC7901 cells were treated with various concentrations of NDEA, NDELA or NDPA for 1 h, followed by drug-free incubations. When the IC_50_ concentrations were compared, the results showed that the cells with the endogenous knock down of BRCA2, caused by RNA interference, exhibited a >2-fold increase in sensitivity to NDELA (Fig. 4B) or NDPA (Fig. 4C) compared with the vector cells. No significant change was observed in the sensitivity to NDEA (Fig. 4A). These results show that BRCA2 confers sensitivity to NOCs.

## Discussion

NOCs are widely distributed in the environment and are recognized as genotoxic agents and possible chemical carcinogens. Their carcinogenicity mainly depends on their genotoxicity (23–27). NDEA, NDELA and NDPA belong to carcinogen groups 2A, 2B and 3, respectively (28–30). In the present study, it was observed that these three NOCs were able to similarly induce time-dependent DSBs in the SGC7901 cells and that the induced DSB repair varied. The DNA damage induced by NDEA, the most potent carcinogen, was observed to not be repaired as efficiently as that caused by NDELA or NDPA. The results suggested that the pathways of NOC-induced DNA damage repair differ and that this may contribute significantly to an NOC’s genotoxicity.

DNA DSBs are the most lethal DNA lesions, posing an almost insurmountable challenge to the cells’ DNA repair machinery. HR is a DSB repair pathway. The HR pathway uses one sister DNA strand as the repair template and repairs DNA DSBs with high fidelity (11,14,37). In order to understand why the repair of NOC-induced DSBs varied and to determine the role of HR in this process, the expression levels of of BRCA2 and RAD51, two key proteins in HR, were evaluated. The SGC7901 cells exhibited a defective HR in response to NDEA treatment and an increased HR due to NDELA or NDPA treatment. The results show that HR has a significant role in the process of NOC-induced DSB repair, which may explain why the DNA damage induced by NDEA is not repaired as efficiently as that caused by NDELA or NDPA and why NDEA possesses the most potent genotoxic-carcinogenicity among the NOCs.

Nonhomologous end joining (NHEJ) is an alternative mechanism for the repair of DSBs (38). This process joins broken chromosome ends in a manner that does not depend on sequence homology and so may not be error free. Although the NHEJ pathway frequently results in minor changes in DNA sequence at the break site, and occasionally the joining of previously unlinked DNA molecules, it is a major contributor to cell survival following the exposure of mammalian cells to agents that cause DSBs. Since the HR repair of the DNA damage induced by NDELA or NDPA did not vary significantly, it is possible that a differential NHEJ response was induced. However, this hypothesis requires further study.

BRCA2 is a key protein involved in HR. Mammalian cells lacking functional BRCA2 are sensitive to DNA damaging agents and are deficient in homology-directed DNA repair (6–8). Genotoxic agents such as mitomycin C have been associated with decreased BRCA2 protein expression (39). BRCA2 is also degraded during the alkyltransferase-mediated DNA repair of DNA adducts (40). The present study showed that the expression of BRCA2 was inhibited by NDEA treatment, but upregulated with NDELA or NDPA. The knock down of BRCA2 impaired the DNA damage repair induced by NDELA or NDPA. The cells with this knock down showed an increased sensitivity to NDELA or NDPA, suggesting that BRCA2 may have a particularly significant role in differential DSB repair in response to NOC-induced DSBs.

NDEA, NDELA and NDPA are three NOCs with similar chemical structures, but different carcinogenic risks. The present study demonstrated that these NOCs had similar effects on DNA damage. NDELA- or NDPA-induced DNA damage was observed to be more effectively repaired than that induced by NDEA. NDELA and NDPA upregulated the expression of BRCA2 and RAD51, but NDEA did not. Furthermore, it was observed that the knock down of BRCA2 blocked NDELA- or NDPA-induced DNA damage repair and also that cells with this knock down showed an increased sensitivity to NDELA or NDPA. Taken together, these observations suggest that the BRCA2-mediated DNA repair pathway may have a significant role in NOC-induced DNA damage repair and that this may be associated with the differential carcinogenicity of these NOCs.
